# Transcutaneous tibial nerve stimulation *versus* parasacral stimulation in the treatment of overactive bladder in elderly people: a triple-blinded randomized controlled trial

**DOI:** 10.6061/clinics/2020/e1477

**Published:** 2020-01-06

**Authors:** Raquel Henriques Jacomo, Aline Teixeira Alves, Adélia Lucio, Patrícia Azevedo Garcia, Dayanne Cristina Ramos Lorena, João Batista de Sousa

**Affiliations:** I Programa de Ciencias Medicas, Faculdade de Medicina, Universidade de Brasilia, Brasilia, DF, BR; II Programa de Pos-Graduacao em Ciencias da Reabilitacao, Departamento de Fisioterapia, Universidade de Brasilia, Brasilia, DF, BR; III Unidade de Reabilitacao, Hospital Universitario, Universidade do Mato Grosso do Sul, Campo Grande, MS, BR

**Keywords:** Overactive Urinary Bladder, Aged, Transcutaneous Electric Nerve Stimulation, Lower Urinary Tract Symptoms

## Abstract

**OBJECTIVES:**

To evaluate the effect of transcutaneous tibial nerve stimulation (TTNS) and transcutaneous parasacral stimulation on the treatment of overactive bladder (OAB) in elderly people and to compare the final results between groups.

**METHODS:**

Fifty female volunteers, mean age 68.62 (±5.9) years, were randomly allocated into two groups: those receiving TTNS (G1, N=25) and those receiving transcutaneous parasacral stimulation (G2, N=25). The primary outcome was the International Consultation on Incontinence Questionnaire (ICIQ-OAB) score, and secondary outcomes were the International Consultation on Incontinence Questionnaire - short form (ICIQ-SF) score and 3-day bladder diary measurements. Volunteers were assessed before and after the treatment. Clinical Trials (ReBeC): RBR-9Q7J7Y.

**RESULTS:**

Both groups’ symptoms improved as measured by the ICIQ-OAB (G1 = <0.001; G2 = <0.001) and ICIQ-SF (G1 = <0.001; G2 = <0.001). In the 3-day bladder diary assessments after treatment, G1 showed a reduced number of nocturia (*p*<0.001), urgency (*p*<0.001) and urge urinary incontinence episodes (*p*<0.001), whereas G2 showed only a reduced number of nocturia episodes (*p*<0.001). No difference between groups was found.

**CONCLUSION:**

Both of the proposed treatments were effective in the improvement of OAB symptoms, but TTNS showed a reduction in a greater number of symptoms as measured by the 3-day bladder diary. No differences were found between groups.

## INTRODUCTION

Overactive bladder (OAB) syndrome is defined as the urgency to void, followed or not by urge urinary incontinence, and it can be associated with symptoms such as daytime micturition frequency and nocturia; the lack of urinary tract infection or other pathology is mandatory for a conclusive diagnosis (1). This condition has an important negative impact on the patient’s quality of life, making the treatment relevant (2). The prevalence of OAB varies by approximately 5 to 50% (3), and symptoms are more likely to happen in elderly people (4). Although OAB is idiopathic, changes induced by aging such as estrogen deprivation (which leads to alterations in bladder function), neurological changes (which lead to changes in bladder sensation, making urgency more perceptive), and anatomic changes (which decrease bladder capacity, detrusor contractility and flow rates) predispose patients to OAB; for this reason, approximately 45% of women aged 65 or more present with the syndrome (5).

The first-line treatment recommended by the International Continence Society (ICS) includes non-invasive methods such as medication, bladder training and pelvic floor muscle training (6). Electrostimulation is a modality of conservative treatment in which the therapeutic mechanism consists of balancing the excitatory and inhibitory impulses that control the bladder; it can be applied in different ways as parasacrally (7), intravaginally/intranally (8) and in the tibial nerve (2).

Tibial nerve stimulation shows encouraging results in the treatment of OAB (2), but, as there is still a lack of published studies of this treatment, its level of recommendation is still confused, and some authors recommend its use as second-line therapy (5) and others third-line therapy (9). The tibial nerve can be stimulated in a percutaneous way, where a needle is positioned directly in the nerve, or in a transcutaneous way, where surface electrodes are positioned in the region of the innervations of the tibial nerve; the latter is used more frequently by physiotherapists because it is a non-invasive method. A recent study showed that both of the described methods have the same results in the treatment of OAB (3).

Sacral stimulation is another way to balance bladder impulses, but this electrostimulation modality has been tested only in children (10) or with implantable electrodes (9). No published data are available on parasacral transcutaneous electrostimulation in the treatment of OAB in adults.

As the particularities of the elderly population must be taken into account in the prognosis of a treatment plan, as tibial nerve stimulation in the treatment of OAB is still scarce in the literature, and as sacral stimulation, which is effective in modulating nervous control of the bladder, has never been tested in the OAB elderly population, the aims of this study are to assess the results of transcutaneous tibial nerve stimulation and parasacral electrostimulation in the treatment of OAB in elderly women and to compare the final results between groups in an attempt to determine whether one modality presents better results than the other in elderly women.

## METHODS

A randomized controlled trial was conducted by the Program of Micturition Dysfunction at a health center in Brasília, Brazil, from August 2017 to October 2018. The study was approved by the Research Ethics Committee of School of Medicine of the University of Brasília (CAEE-55919916.9.0000.5558- December 1, 2016) in accordance with the Declaration of the World Medical Association and Brazilian Registry of Clinical Trials (REBEC), protocol number RBR-9Q7J7Y. Consort statements were used. All patients signed the informed consent form before the beginning of the study.

Participants were eligible for inclusion if they were between 60 and 80 years old and had received a clinical diagnosis of OAB, defined by the presence of urgency followed or not by urge urinary incontinence, nocturia and an increased frequency of daytime urination.

The exclusion criteria were patients with positive urinalysis and urine culture; a history of irradiation and/or hormonal therapy in the last six months; the presence of neurological diseases (multiple sclerosis, Alzheimer’s disease, stroke and Parkinson’s disease); the use of anticholinergic drugs, calcium antagonists, b-antagonists, and dopamine antagonists; the presence of pelvic organ prolapse (POP), as measured by a score greater than III by the POP-Q system; and the inability to answer the questionnaires properly. Clinical and sociodemographic variables such as age, BMI, parity and the number of vaginal deliveries were registered.

Group randomization was performed using a computer program for online randomization (http://www.random.org) by a blind, block-based assessor. Patients were divided into two groups: those receiving transcutaneous tibial nerve stimulation (TTNS; G1) and those receiving transcutaneous parasacral stimulation (G2).

### Main outcome measures

The primary outcome was the score on the International Consultation on Incontinence Questionnaire (ICIQ-OAB) (11). Secondary outcomes were the International Consultation on Incontinence Questionnaire - short form (ICIQ-SF) (12) score and 3-day bladder diary measurements.

All assessments were performed before and after the intervention by a physiotherapist who was unaware of allocation of the volunteers.

The ICIQ-OAB is a brief questionnaire specific to assess OAB symptoms. It comprises 4 questions that measure the impact of symptoms, such as urinary incontinence, urgency, nocturia and daytime micturition frequency, on patients’ lives (11). The score of the questionnaire varies from 0 to 16, and the higher the score, the greater the impact.

The ICIQ-SF is another brief questionnaire that assesses the level and impact of urinary incontinence on patients’ lives. The ICIQ-SF is comprised of 3 questions that assess daytime micturition frequency, the severity of urinary incontinence and the impact of urinary incontinence on quality of life. Furthermore, there are eight questions that assess the possible causes of urinary incontinence. The final score is the sum of the score from three first questions and varies from 0 to 21; the higher the score, the worse the outcome (12).

The 3-day bladder diary accounted for measuring the frequency of urination, nocturia, urgency and urge incontinence during 3 consecutive days. The mean frequency of each symptom over the three days was considered the final result (13).

### Intervention

Using biphasic current and surface electrodes, both groups underwent 8 sessions of 30 minutes of electrical stimulation twice a week using a DUALPEX 961^®^ (Quark, Brazil) electrical stimulation device.

In G1, one surface electrode was applied below the left medial malleolus, and the other electrode was applied 5 cm cephalad to the distal electrode. To confirm the proper electrode position, a frequency of 1 Hz and a pulse width of 200 μs were used, so the presence of rhythmic flexions of the toes during the increase in stimulus intensity was indicative of correct electrode placement. After this procedure, the frequency was increased to 10 Hz, a therapeutic frequency used to treat OAB symptoms (14). The intensity that was used was just below that of the motor response.

For G2, the electrodes were positioned symmetrically in the parasacral region under the posterior superior iliac spines to stimulate nerve roots S2 and S3. A frequency of 10 Hz and a pulse width of 700 μs were used (10). The level of intensity was adjusted according to the patient’s tolerance.

### Statistical analysis

Sample size calculation was based on a pilot study with 10 patients in each group, considering the scores of the first outcome assessment, ICIQ-OAB, and the secondary outcome assessment, ICIQ-SF. The program used for calculation was the *a priori* method in the G*Power software (version 3.1.3; University of Trier, Trier, Germany). Thus, a sample size of 17 and 23 participants, respectively, for G1 and G2 was determined to have a power of 80% and an alpha error of 0.05.

Statistical analysis was performed using the Statistical Package for Social Sciences (SPSS), version 22.0. A third researcher who was blind to the groups’ alignment performed the statistical analysis that followed the principles of per-protocol (PP) analysis. As the data lacked a normal distribution using the Kolmogorov-Smirnov test, continuous data were presented as the median and interquartile range. To compare results within groups, the Wilcoxon test was used, and to compare outcome results between groups, the Mann-Whitney test was used. A *p*-value of 0.05 was considered significant.

## RESULTS

**Figure 1 f01:**
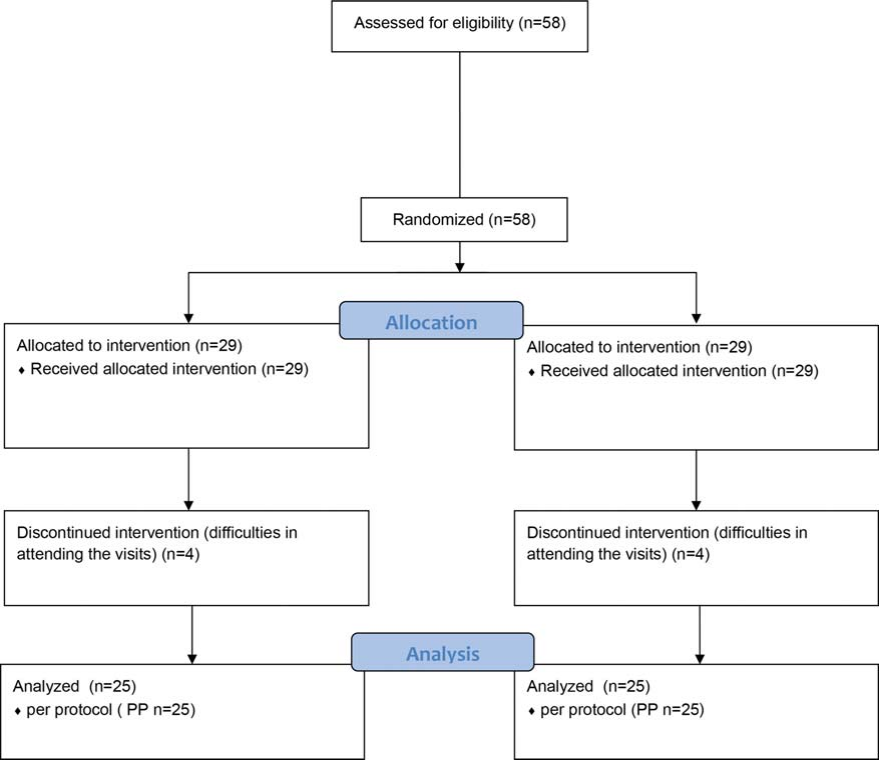
Flowchart.

According to the eligibility criteria, 58 patients were recruited, of whom 8 patients, four in each group, were excluded because they could not attend the physiotherapy sessions. Therefore, 25 patients per group completed the study (Figure 1).

Demographic and initial assessments are shown in Table 1.

As no significant differences were found between groups, assessments were homogeneous at baseline. Median and 25^th^ and 75^th^ interquartile values of demographic and ICIQ-OAB and ICIQ-SF scores and 3-day bladder diary data.

At the end of the treatment, both groups showed significant improvements in the signs and symptoms of OAB, but G1 showed a significant decrease in the number of urgency episodes and urge urinary incontinence episodes, whereas G2 did not show any differences in these assessments. Although more significant findings were found in G1, the improvements shown in the analysis within groups were not enough to show differences between groups. The *p*-values, medians and interquartile ranges of all assessments after the treatment are described in Table 2.

Results of primary and secondary assessments after 8 weeks of treatment. Median (SD) and 25^th^ and 75^th^ interquartile ranges of demographic data, ICIQ-OAB and ICIQ-SF scores and 3-day bladder diary data. There were no differences between the groups. Both groups showed improvement in questionnaire scores. G1 showed a significant decrease in the number of urgency episodes, nocturia and urge urinary incontinence, whereas G2 showed improvement only in the reduction of nocturia episodes.

No side effects were noticed with either the tibial or parasacral therapies.

## DISCUSSION

The present study showed that both of the proposed treatments were effective in the treatment of OAB, whereas the tibial stimulation group showed more significant results than the parasacral stimulation group. Electrode sites for delivering electrical stimulation have different mechanisms of action, but the goal for the final result is the same: to achieve balance in the neural impulses that control the bladder. The advantages and disadvantages of alternative sites of electrostimulation in the treatment of OAB must be tested to offer treatment options to the patients, and to our knowledge, this is the first study that tested the treatment of OAB using parasacral electrostimulation in adults (15).

Although both electrostimulation modalities showed the same results in the majority of the variables at the end of the treatment, the transcutaneous tibial nerve stimulation group showed significant differences in the reduction of urgency and urge urinary incontinence, whereas the parasacral group did not show differences in these variables. Considering that bladder innervations were modulated by the two sites of electrostimulation, we suggest that the reasons for the difference in these results are probably because, when stimulating the tibial nerve, the correct position of the electrodes can be confirmed by the presence of rhythmic flexions of the toes during the increase in the stimulation intensity. On the other hand, the positions of the electrodes for parasacral stimulation are guided by anatomy, and there is no way to confirm whether the nerve roots S2 and S3 are adequately being stimulated; nevertheless, our results show that parasacral stimulation is an alternative route when tibial nerve stimulation is not possible (for example, in the case of leg amputation or peripheral neuropathy).

The findings of this study corroborate those of a previous retrospective study in which 24 elderly patients, with a mean age of 70.25±11.14 years, received 30 minutes of percutaneous tibial nerve stimulation once a week for 12 weeks and showed a reduction of urgency episodes, urge urinary incontinence and nocturia (16). In a previous retrospective study, 62 women with OAB, with a mean age of 72.7 years, underwent 12 sessions of percutaneous tibial nerve stimulation and presented a decrease in urgency and urge urinary incontinence episodes (17); therefore, the electrical stimulation of the tibial nerve seems to be effective in the treatment of OAB in adults and elderly people. Even so, studies should take into account the differences in idiopathic OAB in different age groups as non-elderly people have no plausible reason to develop OAB, and elderly people are likely to have OAB because of anatomic changes caused by age. Aging produces changes in the brain that can induce greater sensation of the urgency to void, and older people can also present bladder ischemia, which can lead to detrusor overactivity or changes in bladder contractility. Another cause of detrusor overactivity in elderly individuals compared to the younger population is the increase in the non-neuronal release of adenosine triphosphate and acetylcholine induced by detrusor muscle stretch. Finally, elderly people have an increased release of inflammatory mediators from the urothelium that can initiate detrusor contractions without changes in neurological control. Furthermore, postmenopausal women have estrogen deprivation, as receptors of this hormone are present in pelvic floor muscles, the bladder urothelium and urethral epithelium, and its deprivation leads to an impairment of bladder functioning (5). Future studies should take age range into account, and further studies are needed to determine which treatment model would achieve better results in the treatment of elderly people.

The limitation of this study is the 8 sessions of treatment, since studies usually treat OAB volunteers for 12 weeks (18). Even so, the information that patients can benefit from a reduced number of sessions is important in terms of the costs of treatment. Furthermore, this study was also limited by the lack of follow-up assessments, making it difficult to draw a conclusion about how long treatment effects will last.

## CONCLUSION

Although there were no differences between groups at the end of the treatment in any assessment, TTNS and parasacral electrostimulation were effective in reducing ICIQ-OAB and ICIQ-SF scores; the TTNS group showed a significant decrease in number of urgency episodes, nocturia and urge urinary incontinence; and parasacral showed improvement only in the reduction of nocturia episodes. [Fig f01]

## AUTHOR CONTRIBUTIONS

Jacomo RH and Lucio A provided substantial contributions to the concept, design, drafting and critical revision of the manuscript for important intellectual content. Alves AT provided substantial contributions to the conception and design. Garcia PA and Lorena DCR contributed to the drafting and critical revision of the manuscript for important intellectual content. de Sousa JB provided substantial contribution to the approval of final version of the manuscript to be published. All authors agreed to be accountable for all aspects of the work to ensure that questions related to the accuracy or integrity of any part of the study are appropriately investigated and resolved and all authors participated sufficiently in the work to take public responsibility for appropriate portions of the content.

## Figures and Tables

**Table 1:** Demonstration of demographic and clinical data (n=50).

Variable	G1	G2	*p*-value
Age (years)	66 (65-73)	66.5 (62.5-75.75)	0.892
Parity (number)	4 (3-6)	4 (3-7.5)	0.577
Vaginal deliveries	2 (1-4)	3 (1.25-5.75)	0.337
ICIQ-OAB score	8.0 (6.7-11.2)	8.0 (6-12)	0.625
ICIQ-SF score	16.5 (15.5-18)	16.0 (13-18)	0.302
**3-day bladder diary**			
Daytime micturition frequency	7.3 (4.1-10)	6.9 (5.5-10.0)	0.481
Nocturia	1.8 (0-1.6)	1.6 (1.2-3.3)	0.877
Number of urgency episodes	1.3 (0-1.6)	1.1 (0-3)	0.814
Number of urge urinary incontinence episodes	1.4 (0-2.5)	1.6 (0.2-3)	0.798

**Table 2:** Changes in the parameters after eight weeks of treatment in TTNS group and Parasacral group by protocol.

Variable	Groups									
	Posterior Tibial TENS (n=25)					Parasacral TENS (n=25)				
	Pre	Post	Mean Difference [95% CI]	*p*-value	Effect Size	Pre	Post	Mean Difference [95% CI]	*p*-value	Effect Size
ICIQ-OAB score	8.64 (3.02) 8 [6.75-11.25]	3.95 (3.07) 3.5 [1.75-7]	4.43 [2.37; 6.48]	<0.001*	1.54	8.88 (2.89) 8 [6-12]	5.95 (4.12) 6 [1.75-9.25]	2.57 [0.63-4.51]	<0,001*	0.80
ICIQ-SF score	16.28 (2.74) 16.5 [15.5-18]	10 (5.70) 8.5 [6.5-16.25]	6.3 [3.46- 9.14]	<0.001*	1.27	15.24 (±3.41) 16 [13-18]	9.67 (7.58) 12.5 [3.75-17.25]	5.33 [1.8-8.87]	<0.001*	0.85
**3-day voiding diary**										
Daytime micturition frequency	6.75 (4.3) 7.3 [4.16-10]	7.20 (2.73) 6.66 [5.32-9.33]	-0.06 [-2.16; 2.03]	0.87	0.11	7.31 (3.9) 6.96 [5.52-10.08]	6.57 (3.68) 6 [4.64-9.62]	0.68 [-0.12-1.49]	0.07	0.2
Number of urgency episodes	1.42 (1.6) 1.33 [0-1.66]	0.77 (1.20) 0 [0-1.49]	0.71 [0.16; 1.27]	<0.001*	0.43	1.60 (1.82) 1.16 [0-3.08]	1.51 (2.41) 0.33 [0-3]	0.10 [-0.80-0.99]	0.373	0.04
Number of urge urinary incontinence episodes	1.92 (2.07) 1.49 [0-2.5]	0.80 (1.37) 0.33 [0-0.74]	1.19 [0.13; 2.25]	<0.001*	0.61	1.83 (2.17) 1.66 [0.25-3]	2.13 (3.28) 1.16 [0-3.41]	-0.23 [-1,59-1,12]	0.84	0.10
Nocturia	2.13 (1.49) 1.83 [0.49-3]	1.51 (1.29) 1.6 [0-2.6]	0.46 [0.10; 0.97]	<0.001*	0.45	2.57 (2.02) 1.66 [1.25-3.32]	1.41 (1.45) 1 [0.25-1.74]	0.93 [0.31-1.55]	<0.001*	0.64

* Wilcoxon test; ICIQ-OAB- International Consultation on Incontinence Questionnaire; ICIQ-SF- International Consultation on Incontinence Questionnaire - short form. G1- Transcutaneous tibial nerve stimulation, G2- parasacral electrostimulation.

## References

[1] Haylen BT, de Ridder D, Freeman RM, Swift SE, Berghmans B, Lee J (2010). An International Urogynecological Association (IUGA)/International Continence Society (ICS) joint report on the terminology for female pelvic floor dysfunction. Int Urogynecol J.

[2] Wibisono E, Rahardjo HE (2015). Effectiveness of Short Term Percutaneous Tibial Nerve Stimulation for Non-neurogenic Overactive Bladder Syndrome in Adults: A Meta-analysis. Acta Med Indones.

[3] Ramírez‐García I, Blanco‐Ratto L, Kauffmann S, Carralero‐Martínez A, Sánchez E (2019). Efficacy of transcutaneous stimulation of the posterior tibial nerve compared to percutaneous stimulation in idiopathic overactive bladder syndrome: Randomized control trial. Neurourol Urodyn.

[4] Potts JM, Payne CK (2018). Urinary Urgency in the Elderly. Gerontology.

[5] Pratt TS, Suskind AM (2018). Management of Overactive Bladder in Older Women. Curr Urol Rep.

[6] Abrams P, Andersson KE, Birder L, Brubaker L, Cardozo L, Chapple C (2010). Fourth International Consultation on Incontinence Recommendations of the International Scientific Committee: Evaluation and treatment of urinary incontinence, pelvic organ prolapse, and fecal incontinence. Neurourol Urodyn.

[7] Veiga ML, Queiroz AP, Carvalho MC, Braga AA, Sousa AS, Barroso U (2016). Parasacral transcutaneous electrical stimulation for overactive bladder in children: An assessment per session. J Pediatr Urol.

[8] Scaldazza CV, Morosetti C, Giampieretti R, Lorenzetti R, Baroni M (2017). Percutaneous tibial nerve stimulation versus electrical stimulation with pelvic floor muscle training for overactive bladder syndrome in women: results of a randomized controlled study. Int Braz J Urol.

[9] Gormley EA, Lightner DJ, Faraday M, Vasavada SP (2015). American Urological Association; Society of Urodynamics, Female Pelvic Medicine. Diagnosis and treatment of overactive bladder (non-neurogenic) in adults: AUA/SUFU guideline amendment. J Urol.

[10] Barroso U, Viterbo W, Bittencourt J, Farias T, Lodêlo P (2013). Posterior tibial nerve stimulation vs parasacral transcutaneous neuromodulation for overactive bladder in children. J Urol.

[11] Pereira SB, Thiel Rdo R, Riccetto C, Silva JM, Pereira LC, Herrmann V (2010). [Validation of the International Consultation on Incontinence Questionnaire Overactive Bladder (ICIQ-OAB) for Portuguese]. Rev Bras Ginecol Obstet.

[12] Tamanini JT, Dambros M, D’Ancona CA, Palma PC, Rodrigues-Netto N (2005). Responsiveness to the Portuguese version of the International Consultation on Incontinence Questionnaire-Short Form (ICIQ-SF) after stress urinary incontinence surgery. Int Braz J Urol.

[13] Jimenez-Cidre MA, Lopez-Fando L, Esteban-Fuertes M, Prieto-Chaparro L, Llorens-Martinez FJ, Salinas-Casado J (2015). The 3-day bladder diary is a feasible, reliable and valid tool to evaluate the lower urinary tract symptoms in women. Neurourol Urodyn.

[14] Bellete PO, Rodrigues-Palma PC, Hermann V, Riccetto C, Bigozzi M, Olivares JM (2009). [Posterior tibial nerve stimulation in the management of overactive bladder: a prospective and controlled study]. Actas Urol Esp.

[15] Quintiliano F, Veiga ML, Moraes M, Cunha C, de Oliveira LF, Lordelo P (2015). Transcutaneous parasacral electrical stimulation vs oxybutynin for the treatment of overactive bladder in children: a randomized clinical trial. J Urol.

[16] Kurdoğlu Z, Carr D, Harmouche J, Ünlü S, Kılıç GS (2018). Short-term results of the efficacy of percutaneous tibial nerve stimulation on urinary symptoms and its financial cost. J Turk Ger Gynecol Assoc.

[17] Rostaminia G, Chang C, Pincus JB, Sand PK, Goldberg RP (2019). Predictors of successful percutaneous tibial nerve stimulation (PTNS) in the treatment of overactive bladder syndrome. Int Urogynecol J.

[18] Garcia MBS, Pereira JS (2018). Electrostimulation of the posterior tibial nerve in individuals with overactive bladder: a literature review. J Phys Ther Sci.

